# Wide-Range Magnetoelectric Response on Hybrid Polymer Composites Based on Filler Type and Content

**DOI:** 10.3390/polym9020062

**Published:** 2017-02-14

**Authors:** Pedro Martins, Marco Silva, Silvia Reis, Nélson Pereira, Harvey Amorín, Senentxu Lanceros-Mendez

**Affiliations:** 1 Centro de Física, Universidade do Minho, 4710-057 Braga, Portugal; pmartins@fisica.uminho.pt (P.M.); marco.uminho@gmail.com (M.S.); nmmsp.18@gmail.com (N.P.); 2 Centro Algoritmi, Universidade do Minho, 4800-058 Guimarães, Portugal; silviacmreis@gmail.com; 3 Instituto de Ciencia de Materiales de Madrid, CSIC, Cantoblanco, 28049 Madrid, Spain; hamorin@icmm.csic.es; 4 BCMaterials, Parque Científico y Tecnológico de Bizkaia, 48160 Derio, Spain; 5 IKERBASQUE, Basque Foundation for Science, 48013 Bilbao, Spain

**Keywords:** magnetoelectric, composite, magnetostrictive, piezoelectric, wide-range magnetic field

## Abstract

In order to obtain a wide-range magnetoelectric (ME) response on a ME nanocomposite that matches industry requirements, Tb0.3Dy0.7Fe1.92 (Terfenol-D)/CoFe_2_O_4_/P(VDF-TrFE) flexible films were produced by the solvent casting technique and their morphologic, piezoelectric, magnetic and magnetoelectric properties were investigated. The obtained composites revealed a high piezoelectric response (≈−18 pC·N^−1^) that is independent of the weight ratio between the fillers. In turn, the magnetic properties of the composites were influenced by the composite composition. It was found that the magnetization saturation values decreased with the increasing CoFe_2_O_4_ content (from 18.5 to 13.3 emu·g^−1^) while the magnetization and coercive field values increased (from 3.7 to 5.5 emu·g^−1^ and from 355.7 to 1225.2 Oe, respectively) with the increasing CoFe_2_O_4_ content. Additionally, the films showed a wide-range dual-peak ME response at room temperature with the ME coefficient increasing with the weight content of Terfenol-D, from 18.6 to 42.3 mV·cm^−1^·Oe^−1^.

## 1. Introduction

Magnetic sensors and energy harvesters have attracted much interest in recent years due to their wide range of applications, which include navigation systems, medical sensors, non-destructive material testing, building monitoring, agriculture management and biomedical areas [1,2,3,4], among others.

Traditional magnetic sensors show important disadvantages, which include the need for a power supply, low spatial resolution, a complex fabrication process, miniaturization problems (for device dimensions on the order of micrometers), high-cost assembly, the need for temperature compensation circuits, large initial offset and reduced accuracy. Furthermore, those devices do not meet increasing industry demands in terms of flexibility, versatility, light weight, cost, complicated shape allowance or low-temperature fabrication processing, hindering their use in novel and rapidly growing application areas such as flexible or wearable devices [3,5].

Polymer-based magnetoelectric (ME) materials are attracting increasing attention as they can solve the above-mentioned problems due to their cheap, facile, scalable and low-temperature fabrication methods, the absence of large leakage currents, the ability to be fabricated in a variety of forms—such as thin sheets or molded shapes—and, in some cases, their biocompatibility [2,5,6,7,8].

ME coefficients on polymer-based ME materials are of the same order of magnitude as the best ones obtained in materials that are already being used/investigated as magnetic sensors and/or energy harvesters. This fact encourages the emergence of a new generation of polymer-based ME devices [9,10]. The ME voltage coefficient, as the figure of merit of a magnetic field sensor, describes the variation of the electric field as a function of the applied magnetic field [3]. However, magnetoelectric composites present strong ME effects only near an optimum direct current (DC) magnetic field, where the effective piezomagnetic coefficient of the magnetostrictive layer is at its maximum, this fact being the main disadvantage of magnetoelectric devices, as it compromises their use in high-sensitivity miniaturized magnetic devices [3].

Trying to solve such limitations, some efforts have been devoted to obtaining a multi-peak ME phenomenon on ME devices such as the one proposed by Chen et al. [3]. In their study, the interaction between Terfenol-D and FeSiB resulted in dual-peak occurrence, the first peak being caused by the strong exchange coupling effect between Terfenol-D and FeSiB layers and the second peak being caused by the maximum of the dynamic piezomagnetic coefficient q_33_ of the Terfenol-D layer. This pioneer report proved that it was possible to tailor and optimize the ME response by combining different magnetostrictive components in the same ME composite. On the other hand, the developed composite was a laminated structure with several drawbacks, such as the effective ME coupling of the (2-2) film connectivity being limited by the clamping of the films to the substrate and detrimental dielectric leakage currents [11]. A possible solution would be the use of nanocomposites, which offer advantages such as higher flexibility, simpler fabrication, easy shaping, miniaturization possibilities, and the absence of degradation at the piezoelectric/magnetostrictive interface [12,13].

Thus, it is scientifically and technologically relevant to obtain a multi-peak ME response on ME nanocomposites to match a material’s properties and responses with the ones suitable for practical applications [3].

In this work, two types of highly magnetostrictive particles, Terfenol-D and CoFe_2_O_4_, were added to a poly(vinylidene-trifluoroethylene) (P(VDF-TrFE)) piezoelectric matrix, aiming to tailor the ME response of polymer-based composites through the variation of the magnetostrictive filler type and content.

Terfenol-D microparticles were selected once they exhibited the highest room-temperature magnetostrictive coefficient (600 ppm) among the microparticles. CoFe_2_O_4_ nanoparticles were selected due to having the highest magnetostriction (≈200 ppm) among ferrite nanoparticles [14,15]. Additionally, the optimum DC magnetic field, where the effective piezomagnetic coefficient of the magnetostrictive particles is maximized, is different for the two particle types, allowing a double-peak phenomenon of the ME response of the Terfenol-D/CoFe_2_O_4_/P(VDF-TrFE) hybrid composite. P(VDF-TrFE) was selected as the piezoelectric matrix due to its highest piezoelectric responses among polymer materials over a wide range of temperatures [9,16].

## 2. Materials and Methods

### 2.1. Materials

N,N-Dimethylformamide (DMF, pure grade) was supplied by Fluka (Milwaukee, WI, USA) and P(VDF-TrFE) was supplied by Solvay Solexis (West Deptford, NJ, USA). CoFe_2_O_4_ nanoparticles were purchased from Nanoamor (Houston, TX, USA) with dimensions between 35–55 nm. Terfenol-D powder with a mean particle size of ≈1 µm was obtained from ETREMA Products, Inc. (Ames, IA, USA). All chemicals were used as received without further purification.

### 2.2. Terfenol-D/CoFe_2_O_4_/P(VDF-TrFE) Composite Preparation

The multiferroic composites were prepared following procedures reported on [2,9,12]. Briefly, the selected filler content of the magnetostrictive phase (Terfenol-D and CoFe_2_O_4_) was added into DMF solvent and placed in an ultrasound bath for 8 h aiming to ensure a good dispersion of the magnetostrictive phase. P(VDF-TrFE) polymer was then added and mixed for 2 h with a Teflon mechanical stirrer in an ultrasound bath to prevent magnetic agglomeration during the mixing process. The, the resulting mixture was spread on a clean glass substrate and solvent evaporation and polymer melting were performed inside an oven for 10 min at 210 °C. P(VDF-TrFE) crystallization was achieved by cooling down the composite films to room temperature (≈25 °C). At the end of the process, the ≈50 µm-thick films were peeled from the glass substrate. Flexible ME composite films were prepared with 40% weight content (wt %) of magnetostrictive filler. It has been shown that for such filler content, the films can be poled without electric breakdown and good ME coupling and flexibility are obtained [12]. To study the influence of each magnetostrictive particle type on the ME response of the developed Terfenol-D/CoFe_2_O_4_/P(VDF-TrFE) nanocomposites, three distinct samples were produced (further refereed in the paper by the name provided in parenthesis): hybrid composites with 10 wt % (0.02 in volume fraction)of Terfenol-D and 30 wt % (0.13 in volume fraction) of CoFe_2_O_4_ (10TD/30CFO); 20 wt % (0.05 in volume fraction) of Terfenol-D and 20 wt % (0.08 in volume fraction) of CoFe_2_O_4_ (20TD/20CFO); and 30 wt % (0.08 in volume fraction) of Terfenol-D and 10 wt % (0.04 in volume fraction) of CoFe_2_O_4_ (30TD/10CFO).

### 2.3. Terfenol-D/CoFe_2_O_4_/P(VDF-TrFE) Composite Characterization

The morphology of the Terfenol-D/CoFe_2_O_4_/P(VDF-TrFE) composites was evaluated via scanning electron microscopy (SEM) with a Quanta 650 FEI scanning electron microscope (Hillsboro, OR, USA) at 10 kV. Before SEM, samples were coated with gold by magnetron sputtering. Further, composition analysis was carried out by energy-dispersive X-ray microanalysis (EDS) from 0 to 13 keV.

In order to optimize the piezoelectric response, poling of the Terfenol-D/CoFe_2_O_4_/P(VDF-TrFE) nanocomposites was performed in a home-made chamber, after an optimization procedure, by corona poling at 10 kV during 120 min at 120 °C and cooling down to room temperature under the applied electric field. The piezoelectric response (d_33_) of the composites was evaluated with a wide range d_33_-meter (model 8000, APC Int Ltd., Mackeyville, PA, USA). Room-temperature magnetic hysteresis loops were measured with a Microsense 2.2 Tesla Vibrating Sample Magnetometer (Lowell, MA, USA) vibrating sample magnetometer (VSM).

The ME coefficient α_33_ was measured with the application of both DC and AC magnetic fields along the direction of the electrical polarization of the composites, i.e., perpendicular to the surface.

The AC driving magnetic field of 1 Oe amplitude at ≈8 kHz (resonance of the Terfenol-D/CoFe_2_O_4_/P(VDF-TrFE) composites) was delivered by a pair of Helmholtz coils and the DC field with a maximum value of 0.5 T was applied by an electromagnet.

The resonance frequency (*f*_r_) of the composites was calculated by using Equation (1):

(1) $$f_{r}=\frac{n}{2t}\sqrt{\frac{E_{Y}}{\rho }}$$

where n, t, E_Y_ and *ρ* are the harmonic mode order, thickness, in-plane Young’s modulus and density of the composites, respectively. The produced ME voltage (ΔV) was measured with a Standford Research Lock-in amplifier (SR530, Sunnyvale, CA, USA). Circular 1.4 mm-diameter gold electrodes were sputtered on the opposite sides of the samples prior to the ME characterization.

The ME coefficient α_33_ was determined through Equation (2):

(2) $$\alpha_{33}=\frac{\Delta V}{t\times B_{AC}}$$

where ∆V is the ME voltage generated in the composite, B_AC_ the AC magnetic field and t the thickness of the ME composite.

## 3. Results and Discussion

**Figure 1:**
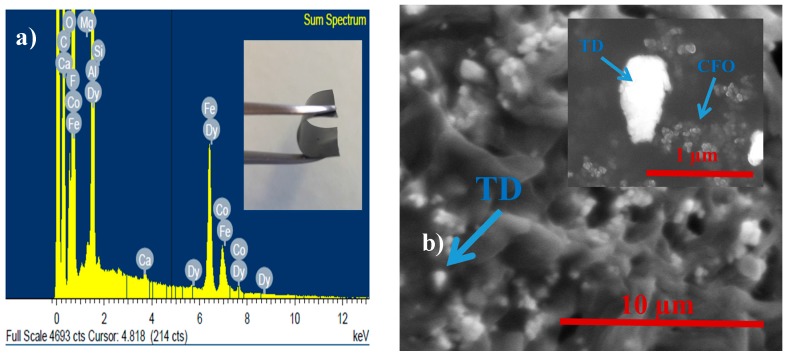
(**a**) EDS analysis of the 20TD/20CFO composite (inset reveals a photograph of such a flexible composite); and (**b**) SEM image showing the TD dispersion on the 20TD/20CFO composite as a magnification showing both magnetostrictive particles (inset).

After the flexible samples, such as the one represented in the inset of Figure 1a, were obtained, SEM images were taken in order to verify the dispersion and distribution of the magnetostrictive particles inside the P(VDF-TrFE) matrix.

Additionally, the data in Figure 1a prove the joint presence on the composites of elements of both magnetostrictive particles, Tb, Dy and Fe from TD and Co, and Fe and O from CFO.

Figure 1b reveals a good distribution of both particle types inside the polymer. Such a good distribution was also observed in the other composite compositions (10TD/30CFO and 30TD/10CFO—images not shown). Additionally, the different size range of TD and CFO fillers was evidenced.

**Figure 2:**
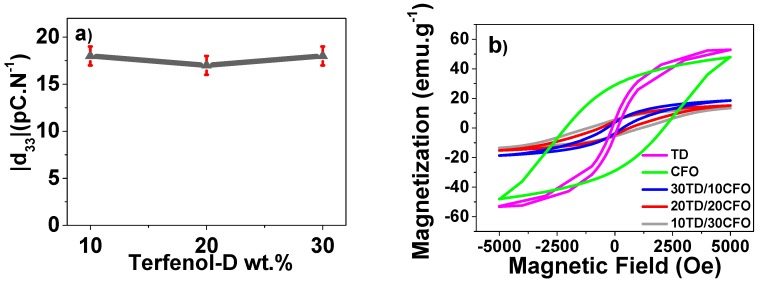
(**a**) Variation of the modulus of the piezoelectric response, the |d_33_| value, as a function of TD/CFO/P(VDF-TrFE) composite composition; (**b**) magnetic response of the TD/CFO/P(VDF-TrFE) composites.

Once the ME response of the TD/CFO/P(VDF-TrFE) composite emerged from the strain-mediated coupling between the piezoelectric and magnetic responses, the effect of the filler content and type on these responses was evaluated, as shown in Figure 2.

Figure 2a shows that the introduction of magnetic fillers on the polymer matrix led to a small decrease in the piezoelectric response (≤20%; 18 pC·N^−1^) when compared to the piezoelectric response of neat P(VDF-TrFE) (−22 pC·N^−1^). This fact is attributed to the disruption of the polymer matrix, in particular at the interfaces with the fillers. Nevertheless, such a piezoelectric response is still suitable for obtaining high ME coefficients in polymer nanocomposites.

Magnetic measurements at room temperature (Figure 2b) allowed us to obtain the magnetic behavior of such composites and compare them with the pure powders (TD and CFO) (Table 1).

It is noted that the *M*_S_ value decreased with the increasing CFO content (from 18.5 to 13.3 emu·g^−1^) once the CFO powder had a lower *M*_S_ (47.8 emu·g^−1^) when compared to the TD powder (52.9 emu·g^−1^). On the contrary, the *M*_R_ and *H*_C_ values increased (from 3.7 to 5.5 emu·g^−1^ and from 355.7 to 1225.2 Oe, respectively) with the increasing CFO content, once CFO had higher *M*_R_ and *H*_C_ values (28.8 emu·g^−1^ and 2100.3 Oe) when compared to the TD powder (4.9 emu·g^−1^ and 117.5 Oe). Results from Table 1 also reveal that the coexistence of both magnetostrictive particles on the same polymeric composite did not hinder the overall magnetic response.

**Figure 3:**
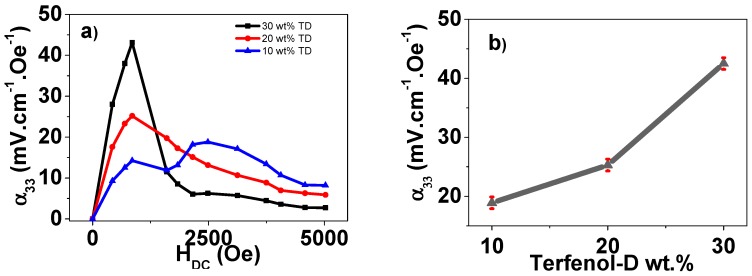
(**a**) ME voltage coefficient (α_33_) as a function of H_DC_ for the Terfenol-D/CoFe_2_O_4_/P(VDF-TrFE) composites; (**b**) variation of the Terfenol-D/CoFe_2_O_4_/P(VDF-TrFE) highest α_33_ value as a function of composite composition.

The appropriate piezoelectric and magnetic responses of the composites being proved, the dependence of the resonant ME voltage coefficient for the TD/CFO/P(VDF-TrFE) composites with the DC bias magnetic field and Terfenol-D content is presented in Figure 3.

Due to the magnetostrictive properties of the fillers, the maximum ME response of the TD/P(VDF-TrFE) and CFO/P(VDF-TrFE) hybrid composites usually takes place at 800–1200 Oe and 2000–3000 Oe magnetic field ranges, respectively [17].

In the composite with a lower CFO content, 30TD/10CFO, the ME voltage peak was almost entirely derived from the TD magnetostrictive phase, although another hump is observable in the 2200–3600 Oe field range.

In the 20TD/20CFO composite, a ME response with a broad peak as a result of the magnetostrictive properties of both TD and CFO fillers was verified. The 10TD/30CFO composite revealed a double-peak with maximum output voltages at the H_DC_ at which the magnetostrictive coefficient of each nanoparticle type was saturated, with 850 and 2500 Oe for TD and CFO, respectively [12].

Due to the higher magnetostrictive coefficient of TD as compared to CFO (600 and 200 ppm, respectively), the composite with the higher content of TD particles reached a higher ME response (Figure 3b) than the one with the higher CFO content (30 and 18 mV·cm^−1^·Oe^−1^, respectively).

Such results demonstrate that it is possible to tailor the ME response of the nanocomposites by combining different magnetostrictive fillers in the same composite, allowing the fabrication of high-sensitivity miniaturized magnetic devices [3]. Additionally, such a non-single-peak ME response is also useful for energy-harvesting devices once it allows a larger energy-harvesting performance in a broader magnetic field range.

## 4. Conclusions

Nanocomposite films based on highly magnetostrictive CFO nanoparticles and TD microparticles dispersed in a piezoelectric P(VDF-TrFE) matrix were prepared by solvent casting with an overall filler content ≈40 wt %. The obtained multiferroic nanocomposites revealed a stable piezoelectric response (≈−18 pC·N^−1^) that is independent of the weight ratio between the fillers. The magnetization saturation values decrease (from 18.5 to 13.3 emu·g^−1^), whereas the remanent magnetization and coercive field values increase (from 3.7 to 5.5 emu·g^−1^ and from 355.7 to 1225.2 Oe, respectively) with the increasing CFO content.

Additionally, these films showed a strong ME coupling at room temperature with the ME coefficient increasing with the TD content up to 42.3 mV·cm^−1^·Oe^−1^, for the sample with 30 wt %. As compared to films with just one magnetostrictive filler, the developed polymer-based composite films showed a double-peak wide-range ME response, together with the highest ME response found on polymer-based particulate composites.

## Figures and Tables

**Table 1:** Magnetic properties (magnetization saturation at 5000 Oe: *M*_S_; remanent magnetization: *M*_R_ and coercive field: *H*_C_).

Sample	*M*_S_ (emu·g^−1^)	*M*_R_ (emu·g^−1^)	*H*_C_ (Oe)
TD powder	52.9	4.9	117.5
CFO powder	47.8	28.8	2100.3
30TD/10CFO	18.5	3.7	355.7
20TD/20CFO	15.1	4.6	648.1
10TD/30CFO	13.3	5.5	1225.2
